# Special Issue: Islet Inflammation and Metabolic Homeostasis

**DOI:** 10.3390/metabo11020077

**Published:** 2021-01-28

**Authors:** Susan J. Burke, J. Jason Collier

**Affiliations:** 1 Laboratory of Immunogenetics, Pennington Biomedical Research Center, Baton Rouge, LA 70808, USA; 2 Laboratory of Islet Biology and Inflammation, Pennington Biomedical Research Center, Baton Rouge, LA 70808, USA; 3 Department of Biological Sciences, Louisiana State University, Baton Rouge, LA 70803, USA

This special issue was commissioned to offer a source of distinct viewpoints and novel data that capture some of the subtleties of the pancreatic islet, especially in relation to adaptive changes that influence metabolic homeostasis. As such, this forum provided a home for review articles that offered original perspectives relevant to islet inflammation, tissue resident immune cells, and related variables that influence these factors. In addition, we are pleased to note that articles in this issue also included primary data addressing rapidly evolving and exciting areas of research. Indeed, the pancreatic islet is a magnificent micro-organ that is capable of rapid adaptive responses to support the changing needs of the organism [1].

A very timely study by Piñeros et al. shows the impact of one week of high-fat feeding on changes at the single-cell level in mouse islets [2]. This is important for two reasons: (1) after several months on a high-fat diet, islet adaptations (e.g., increases in insulin-positive cell mass, etc.) have already taken place [3,4]. Thus, investigating early time points is essential to understand the initial alterations required for adaptive responses. (2) Outcomes associated with increases in beta-cell mass, such as enhanced proliferation markers, arise early (within days) after a stimulus that promotes insulin resistance (e.g., high-fat feeding, glucocorticoid exposure, etc.) [4,5,6]. Consequently, an analysis of individual cell populations and how these cells cluster into groups one week after high-fat feeding is important complementary information that offers a window into the individual cell level during this adaptive response. The authors found that not all islet β-cells responded the same to one week of high-fat feeding. Indeed, the data were binned into three major clusters and seven minor clusters. The greatest differences were identified within the minor clusters. This fits with overall β-cell heterogeneity and provides unique insight into early β-cell responses prior to the development of overt glucose intolerance and measurable insulin resistance.

Discovering the signaling pathways that promote an increase in β-cell mass as well as those that enhance insulin secretion reflect ongoing research efforts. How macrophages contribute to such alterations in the islet is an important consideration for adaptive responses to insulin resistance and perhaps also to autoimmunity. A review by Jensen and colleagues offers insights into the dual role that macrophages may have in islet biology [7]. Macrophages are derived from circulating monocytes and also arise through conditioning in the tissue where they ultimately reside [8]. This underscores an important role for macrophages to not only support the growth of β-cells developmentally [9,10], but also the probability that they support the growth and function of mature adult β-cells during adaptive responses [11,12].

Locatelli and Mulvihill put forth a viewpoint on the role of low carbohydrate (<30% kcal) or ketogenic (<10% kcal) diets on three specific parameters: islet health with a focus on the preservation of beta-cell mass in models of rodent obesity and diabetes, secretion of islet hormones to maintain glucose homeostasis, and the response of peripheral tissues to insulin. The effects of such diets in pre-clinical studies and clinical outcomes were carefully considered [13]. Furthermore, the translational relevance of low carbohydrate and ketogenic diets is clear when comparing the impact of reducing carbohydrates in settings of T1D and T2D [13,14]. 

With the knowledge that diets are linked to inflammation and islet β-cell dysfunction, several reviews in this Special Issue focus on stimuli that promote islet β-cell inflammation as well as the outcomes associated with oxidative stress and ER stress. Two reviews by M. Cerf provide complementary information on the glucose and lipid toxicity (sometimes referred to collectively as glucolipotoxicity) contribution to dysfunction in β-cells [15,16]. A third review provides an in-depth perspective on oxidative stress from the viewpoint of the NADPH oxidase superfamily [17]. This is particularly important considering how immune cell-derived cytokines have been shown to promote nitrosative and oxidative stress in β-cells [18,19] and the possible linkage between Nox2 and nutrient stress [20]. Moreover, whether glucose is the major culprit, as opposed to fatty acids, is also relevant and raises important questions for the field as a whole to consider [21]. The reviews in this Special Issue offer additional perspectives towards that discussion. Indeed, with β-cells having a high metabolic rate and being a major site of glucose metabolism [22], a deeper understanding of the role of oxidative, nitrosative, inflammation-based, and ER stress is essential to the goal of enhancing proliferation for therapeutic purposes as well as preventing losses in insulin secretion that lead to diabetes.

When contemplating the role of fatty acids in diabetes and islet dysfunction, it should be noted that such lipids come in many forms, including differing degrees of saturation as well as chain lengths (i.e., short, medium, long, etc.). The role of lipids is complex, with discrete species viewed as pro-inflammatory while others have been attributed as having anti-inflammatory properties [23,24,25]. An interesting contribution by the Kimple laboratory explores fatty acids that activate the EP3 receptor, an important G-protein coupled receptor that responds to the arachidonic acid metabolite prostaglandin E_2_ (PGE_2_) as its primary ligand. In this study, the authors used the Black and Tan BRachyury (BTBR) mice homozygous for the *ob/ob* mutation (leptin-deficient) and found there was a differential effect of diet, when combined with BTBR*^ob^* genotype, that impacted the onset of hyperglycemia [26]. In addition, the authors found that changes in gut microbiota were linked with hyperglycemia versus normoglycemia. Moreover, an altered interleukin-1 and PGE^2^/EP3 signaling link with changes in islet β-cells in this mouse model was observed. 

Taken together, the articles in this issue provide unique insights into islet inflammation and metabolic homeostasis. As guest editors, we are grateful for the quality of articles and the discrete and complementary nature of the work contributed to this Special Issue. We look forward to the continued advancement of knowledge in the field of islet biology that builds from the studies presented here. We want to thank the peer reviewers who provided the rigorous scientific evaluation of each submission as well as the members of the Metabolites Editorial Office for their support during the development, review process, and final steps needed to complete this issue of Metabolites.
